# Is adjuvant chemotherapy necessary for early gastric cancer?

**DOI:** 10.20892/j.issn.2095-3941.2020.0636

**Published:** 2021-06-23

**Authors:** Yu Mei, Tienan Feng, Min Yan, Zhenggang Zhu, Zhenglun Zhu

**Affiliations:** 1 Department of General Surgery, Shanghai Key Laboratory of Gastric Neoplasms, Shanghai Institute of Digestive Surgery, Ruijin Hospital, Shanghai Jiao Tong University School of Medicine, Shanghai 200025, China; 2 Clinical Research Institute, Shanghai Jiao Tong University School of Medicine, Shanghai 200025, China

**Keywords:** Early gastric cancer, adjuvant chemotherapy, treatment strategy

## Abstract

**Objective:**

To quantify the potential benefit of adjuvant chemotherapy (ACT) with respect to survival, and to identify factors for predicting prognoses in early gastric cancer patients.

**Methods:**

Patients with pT1 gastric cancer (GC) who underwent radical resection with D2 lymphadenectomy were retrospectively analyzed. Based on lymph node metastasis (LNM) status and treatment regimens, patients were classified into groups, and clinicopathological variables, overall survival (OS), and disease-specific survival (DSS) were compared.

**Results:**

Of 1,050 enrolled patients, 151 patients (14.4%) had a positive LNM status. Submucosal invasion, undifferentiated state, tumor size > 2 cm, ulceration, and lymphovascular invasion were independent risk factors for LNM using multivariate analyses. The 5-year OS of all patients was 96.4%. HER2 positive, perineural invasion, and LNM were independent factors for worse survival. Patients with pT1N3 GC had a worse 5-year OS and DSS than pT1N0, pT1N1, and pT1N2 patients (*P* < 0.001). The 5-year OS and DSS for pT1N1 patients showed no significant difference between ACT and surgery only patients. For pT1N2 patients, the 5-year OS and DSS showed no significant difference between S-1 and Xelox treatments. For pT1N3 patients, 7 (36.8%) received S-1, while 12 (63.2%) received Xelox treatment. Patients receiving Xelox treatment showed a better 5-year OS (75.0% *vs.* 14.3%) and DSS (81.8% *vs.* 20.0%) than patients receiving S-1 (*P* < 0.05).

**Conclusions:**

Curative surgery only was adequate for patients with pT1N0 and pT1N1. Xelox showed no survival benefits for pT1N2 patients. Therefore, S-1 is the optimal choice for pT1N2 patients, when considering adverse effects. Xelox is recommended for pT1N3 patients.

## Introduction

Gastric cancer (GC) ranks as the fifth most commonly diagnosed cancer and third major cause of cancer-caused mortality worldwide^1^. As a multifactorial disorder, the occurrence and development of GC are affected by both genetic and environmental factors, and approximately 50% of cancer events may be caused by environmental factors^2^. Dietary factors (salted foods/nitrites, etc.) and *Helicobacter pylori* infection are the main causes of GC^3^. Although the incidence of GC has decreased over the years due to advances in early screening strategies, the mortality percentage has not changed^4^. Complete resection offers the only chance of curing GC; however recurrence is common^5^. With the development of medical technologies and the wide use of endoscopy, GC is increasingly detected at an early stage, and the management of early GC (EGC) has become more important. EGC is regarded as cancer that is limited to the mucosa or submucosa, with no consideration of lymph node status, and is generally considered to have a good prognosis with a 5-year OS after curative surgery of more than 90%. However, recurrence occurs in 1.79%–8% of patients^6–9^.

According to the National Comprehensive Cancer Network (NCCN) Guidelines (version 1.2020, GC), treatment for patients with pT1N+ GC should include adjuvant chemotherapy (ACT) after curative resection^10^. Postoperative ACT is not recommended for patients with pT1 GC according to the Japanese Gastric Cancer Treatment Guidelines 2018 (5th edition)^11^. In addition, the Chinese Society of Clinical Oncology (CSCO) Guidelines (version 1.2018, GC) suggest that postoperative ACT should be performed in patients with lymph node metastasis (LNM) to reduce recurrence, although there is insufficient empirical medical evidence of the need for postoperative ACT for stage I GC^12^. Postoperative ACT for GC has long been controversial, and postoperative ACT has been shown to tend towards reducing the risk of death in GC, when compared with surgery alone^13^. However, the beneficial role of ACT in patients with EGC has been less reported, and it remains unclear whether postoperative ACT is necessary for EGC.

In an ACTS-GC trial^14^, postoperative S-1 monotherapy was shown to significantly improve survival of stage II/III (excluding pT1) GC patients who suffered D2 or more extensive lymph-node dissection with R0 surgery. The CLASSIC trial^15^ demonstrated the efficacy of adjuvant Xelox, although there were only 11 (1.1%) patients with pT1 GC enrolled in the study.

In the present study, data of pT1 GC patients who underwent radical resection with D2 lymphadenectomy were retrospectively analyzed to quantify the potential benefit of postoperative ACT versus surgery alone, in terms of OS and disease-specific survival (DSS), and to determine the role of postoperative therapy (S-1 monotherapy, and Xelox doublet chemotherapy) as well as factors predicting the prognoses of pT1 GC patients.

## Materials and methods

### Patients

We retrospectively analyzed pT1 GC patients who underwent radical resection with D2 lymphadenectomy in our organization from January 2011 to May 2015. All included patients were histologically confirmed by pathologists. Patients were excluded from the current study based on the following criteria: (i) < 15 lymph nodes harvested; (ii) prior gastric surgery, distant metastasis, other primary malignancies, R1 or R2 surgical margins, or hospital stays exceeding 30 days; (iii) patients who received neoadjuvant chemotherapy or radiotherapy, or who died within 30 days after surgery; and (iv) patients who were lost to follow-up or terminated ACT for any reason. Finally, this study included 1,050 patients for the subsequent analysis. The Ethics Committee of our organization approved the study. Ethical approval was obtained from the Ruijin Hospital Ethics Committee, Shanghai Jiao Tong University School of Medicine, China (No. 2018-151).

### Evaluation of clinicopathological variables

Patient demographics and clinicopathological information were obtained from medical records, including gender, age at surgery, tumor size and location, gross types (ulcerated or non-ulcerated), degree of differentiation, histopathological types, LNM status, number of examined lymph nodes, invasive depth, lymphovascular and perineural invasion, human epidermal growth factor receptor 2 (HER2) status, and postoperative therapy. No personal information was disclosed during the data collection. Tumor location refers to the upper/middle/lower third of the stomach according to the longitudinal position of the center of the tumor. Histopathological types of tumors were classified according to the Japanese classification of gastric carcinoma (3rd English edition)^16^. In this classification, GC is classified into categories, including papillary adenocarcinoma (Pap), well differentiated and moderately differentiated tubular adenocarcinoma (Tub), poorly differentiated adenocarcinoma (Por), signet ring cell carcinoma (Sig), and mucinous adenocarcinoma (Muc). In general, Pap and Tub types are considered differentiated, while Por, Muc, and Sig types are considered undifferentiated. The depth of invasion and LNM status were determined according to the UICC/AJCC TNM staging system for GC patients (8th edition)^17,18^. Cancer cells appearing in endothelial cells arranged in the tubular space or in a vessel wall structure were considered “lymphovascular invasion”. Cancer cells appearing in the perineural space of nerves were considered “perineural invasion”. HER2 positive was diagnosed with IHC 3+ or IHC 2+/FISH+, and HER2 negative was determined with IHC 0, IHC 1+, or IHC 2+/FISH-^19^.

### Treatments after surgery

Observation and postoperative ACT with S-1 or with capecitabine + oxaliplatin (Xelox) were the treatments for patients after surgery. OBS refers to no antitumor therapy following surgery, unless there was a confirmed relapse, and the first-line treatment was administered when relapse was observed. Patients who received ACT were identified based on the preference of surgeons or oncologists, and the ACT was administered within 4 weeks after surgery. Postoperative ACT with S-1 was administered based on the standard protocol of the ACTS-GC: twice daily oral S-1 (40 mg/m^2^) for 4 weeks with a 2-week rest, and repetition of this 6-week cycle within the first year after operation. Postoperative ACT with Xelox was administered according to the following protocol: six 3-week cycles of oral capecitabine (1,000 mg/m^2^ twice daily on days 1–14 of every cycle) combined with intravenous oxaliplatin (130 mg/m^2^ on day 1 of every cycle). Dose reduction or interruption was allowed if patients had toxic effects of grade 3 or 4. The Common Terminology Criteria for Adverse Events (version 4.0) was used to evaluate adverse events.

### Follow-up

Patients were revisited every 2 months within 1 year after surgery. Physical examinations, blood and tumor marker analyses, and computed tomography examinations of the abdomen and pelvis, as well as endoscopy, were administered every 6 months. All patients were followed-up for at least 5-years after surgery or until death or censoring date.

### Statistical analysis

Statistical analyses were conducted using SPSS statistical software for Windows, version 22.0 (IBM, Armonk, NY, USA). Chi-squared or Fisher’s exact tests were used to compare categorical variables, which are shown as numbers with percentages. The distribution of continuous variables was evaluated using normality tests and univariate analyses. Median values with interquartile range were used to present non-normally distributed continuous data. Continuous variables were converted to binary variables, and the median value was selected as the cut-off. Patients were categorized into LNM (+) and LNM (−) according to the LNM status, followed by logistic regression analysis to identify the risk of LNM. Variables with statistical significance using univariate analysis were further analyzed using multivariate analyses. The hazard ratios (HRs) and confidence intervals (CIs) in the multivariate analysis were calculated using Cox proportional-hazards regression. DSS was considered as the phase from surgery to GC-caused death. The 5-year OS and DSS were evaluated using the Kaplan-Meier method with log-rank tests. A value of *P* < 0.05 was regarded as statistically significant.

## Results

### Clinicopathological features of EGC patients

Clinicopathological features of the enrolled patients are shown in **Table 1**. The 1,050 patients with a median age of 60 years all met the criteria for evaluation, of which 66.3% were male (*n* = 696). The median tumor size was 2 cm, and tumors ≤ 2 cm in size accounted for 66.5%. The majority of patients had tumors with lower locations (62.7%), non-ulcerated (67.6%), and with undifferentiated histopathology (63.6%). Some patients (*n* = 131) were HER2 positive, accounting for 12.5%. A total of 151 patients had positive LNM status, accounting for 14.4%. The mean and median numbers of examined lymph nodes were 25 and 21, respectively, with a range from 16 to 91. As of May 2020, the mean and median follow-up times of all patients were 82.0 and 83.2 months, respectively, with a range from 2 to 112.5 months.

**Table 1:** Clinicopathological characteristics of patients with early gastric cancer

Variables	Total (*n* = 1,050)	LNM (−) (*n* = 899)	LNM (+) (*n* = 151)	*P*
Age (years)	60 (52,67)	60 (52,67)	58 (50,67)	0.262
< 60	512 (48.8%)	432 (48.1%)	80 (53.0%)	
≥ 60	538 (51.2%)	467 (51.9%)	71 (47.0%)	
Gender				0.015*
Male	696 (66.3%)	609 (67.7%)	87 (57.6%)	
Female	354 (33.7%)	290 (32.3%)	64 (42.4%)	
Tumor size (cm)	2.0 (1.2,2.5)	2.0 (1.0,2.5)	2.0 (1.5,3.0)	0.002*
≤ 2	698 (66.5%)	614 (68.3%)	84 (55.6%)	
> 2	352 (33.5%)	285 (31.7%)	67 (44.4%)	
Tumor location				0.998
Upper	70 (6.7%)	60 (6.7%)	10 (6.6%)	
Middle	322 (30.7%)	276 (30.7%)	46 (30.5%)	
Lower	658 (62.7%)	563 (62.6%)	95 (62.9%)	
Gross type				< 0.001*
Ulcerated	340 (32.4%)	264 (29.4%)	76 (50.3%)	
Non-ulcerated	710 (67.6%)	635 (70.6%)	75 (49.7%)	
Degree of differentiation				< 0.001*
Differentiated	382 (36.4%)	356 (39.6%)	26 (17.2%)	
Undifferentiated	668 (63.6%)	543 (60.4%)	125 (82.8%)	
Histopathology				< 0.001*
Tub	381 (36.3%)	355 (39.5%)	26 (17.2%)	
Por	490 (46.7%)	389 (43.3%)	101 (66.9%)	
Sig	160 (15.2%)	140 (15.6%)	20 (13.2%)	
Muc	18 (1.7%)	14 (1.6%)	4 (2.6%)	
Pap	1 (0.1%)	1 (0.1%)	0	
Examined lymph node	21 (17,29)	21 (17,28)	23 (18,32)	0.017*
Her-2 status				0.268
Negative	919 (87.5%)	791 (88.0%)	128 (84.8%)	
Positive	131 (12.5%)	108 (12.0%)	23 (15.2%)	
Depth of tumor invasion				< 0.001*
T1a	512 (48.8%)	485 (53.9%)	27 (17.9%)	
T1b	538 (51.2%)	414 (46.1%)	124 (82.1%)	
Lymphovascular invasion	84 (8.0%)	53 (5.9%)	31 (20.5%)	< 0.001*
Perineural invasion	12 (1.1%)	6 (0.7%)	6 (4.0%)	< 0.001*
Treatments after surgery				< 0.001*
Observation	945 (90.0%)	899 (100%)	46 (30.5%)	
S-1	60 (5.7%)	0	60 (39.7%)	
Xelox	45 (4.3%)	0	45 (29.8%)	

*P < 0.05 was considered statistically significant. LNM, lymph node metastasis; Tub, tubular adenocarcinoma; Por, poorly differentiated adenocarcinoma; Sig, signet-ring cell carcinoma; Muc, mucinous adenocarcinoma; Pap, papillary adenocarcinoma; T1a, mucosal invasion; T1b, submucosal invasion.

Clinicopathological characteristics were evaluated by LNM status (negative LNM and positive LNM). No significant differences were detected with respect to age, tumor location, or HER2 status between the 2 groups. Using univariate analysis, several variables including female sex, tumor size > 2 cm, ulcerated gross types, undifferentiated types (especially poor histopathology), more examined lymph nodes, submucosal invasions (T1b), lymphovascular invasions, and perineural invasions tended to be associated with LNM in patients with pT1 GC. These variables were then included in multivariate analyses to identify independent risk factors for LNM. Submucosal invasion (T1b) had the highest odds ratio (OR) of 3.941 (95% CI: 2.486–6.249; *P* < 0.001) in multivariate analysis, followed by undifferentiated, with an OR of 2.834 (95% CI: 1.791–4.483; *P* < 0.001). Other statistically significant variables included tumor size > 2 cm (OR: 1.547; 95% CI: 1.065–2.247; *P* = 0.022), ulceration (OR: 1.605; 95% CI: 1.101–2.341; *P* = 0.014), and lymphovascular invasion (OR: 2.173; 95% CI: 1.302–3.626; *P* = 0.003). These 5 variables were considered as independent risk factors for LNM in patients with pT1 GC (**Table 2**).

**Table 2:** Multivariate logistic regression analyses of lymph node metastases of early gastric cancer patients

Variables	Multivariate analysis		
	*P*	Odds ratio	95% CI
Tumor size > 2 cm	0.022	1.547	1.065–2.247
Ulceration	0.014	1.605	1.101–2.341
Undifferentiated	< 0.001	2.834	1.791–4.483
Submucosal invasion	< 0.001	3.941	2.486–6.249
Lymphovascular invasion	0.003	2.173	1.302–3.626

### Long-term outcomes and predictors of survival of EGC patients

**Figure 1:**
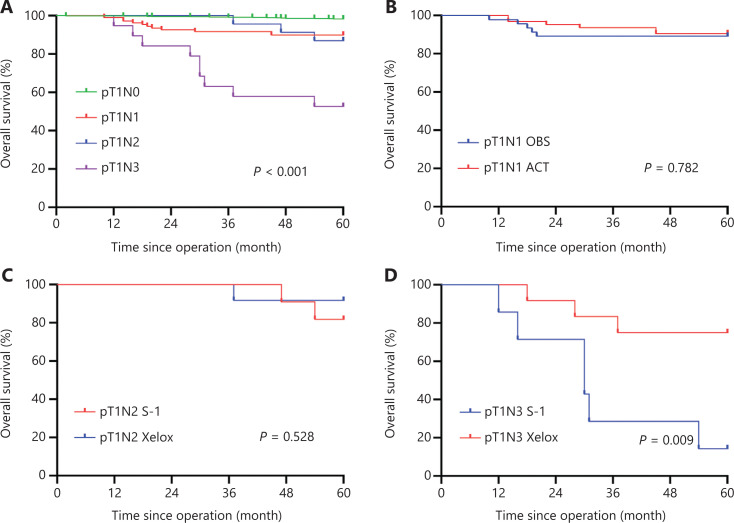
The 5-year overall survival (OS) for pT1 gastric cancer (GC) patients. (A) Kaplan-Meier curves showing the 5-year OS of pT1N0, pT1N1, pT1N2, and pT1N3 GC patients. (B) Kaplan-Meier curves showing the 5-year OS of pT1N1 GC patients in the ACT and OBS groups. (C) Kaplan-Meier curves showing the 5-year OS of pT1N2 GC patients receiving S-1 and patients receiving Xelox. (D) Kaplan-Meier curves showing the 5-year OS of pT1N3 GC patients receiving S-1 and patients receiving Xelox. ACT, adjuvant chemotherapy; OBS, observation.

**Figure 2:**
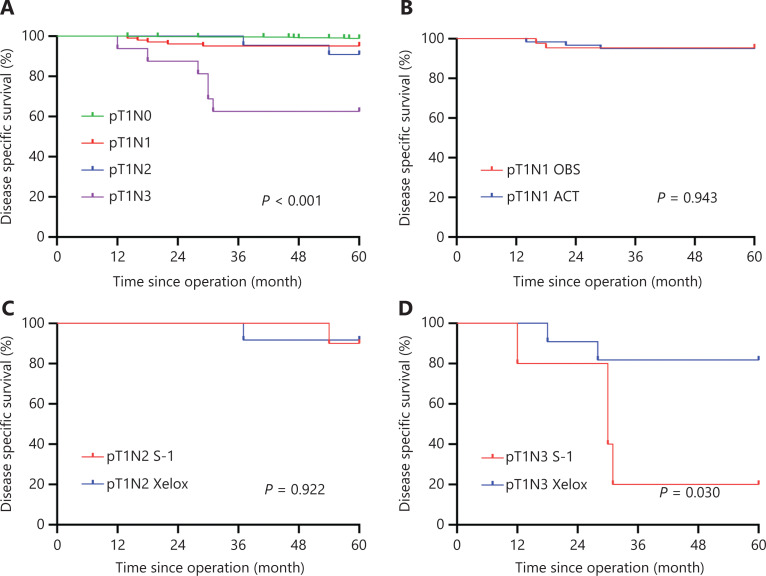
The 5-year disease-specific survival (DSS) for pT1 gastric cancer (GC) patients. (A) Kaplan-Meier curves showing the 5-year DSS of pT1N0, pT1N1, pT1N2, and pT1N3 GC patients. (B) Kaplan-Meier curves showing the 5-year DSS of pT1N1 GC patients in the ACT and OBS groups. (C) Kaplan-Meier curves showing the 5-year DSS of pT1N2 GC patients receiving S-1 and patients receiving Xelox. (D) Kaplan-Meier curves showing the 5-year DSS of pT1N3 GC patients receiving S-1 and patients receiving Xelox. ACT, adjuvant chemotherapy; OBS, observation.

EGC patients had a favorable prognosis with a 5-year OS of 96.4%. According to the UICC/AJCC TNM staging system for GC (8th edition), there were 899 pT1N0, 109 pT1N1, 23 pT1N2, and 19 pT1N3 (including 15 pT1N3a and 4 pT1N3b) GC patients. Patients with pT1N0 GC showed a 5-year OS of 98.3%, while 5-year OS percentages of 89.9% and 87.0% were found for patients with pT1N1 GC and pT1N2 GC, respectively. Patients with pT1N3 GC showed a relatively poor prognosis with a 5-year OS of 52.6% (**Figure 1A**). Using univariate analyses for clinicopathological characteristics with prognosis, depth of tumor invasions, LNM status, lymphovascular invasions, perineural invasions, and HER status had a statistically significant effect on the prognosis. Further multivariate analyses showed that positive LNM had the HR of 6.781 (95% CI: 3.347–13.742; *P* < 0.001), followed by perineural invasion (HR: 4.035; 95% CI 1.122–14.506; *P* = 0.033), and HER2 positive (HR: 3.082; 95% CI: 1.512–6.281; *P* = 0.002). These 3 statistically significant variables were independent factors for worse prognoses in pT1 GC patients (**Table 3**). DSS was also analyzed, except for patients who died from other factors. Similarly, patients with pT1N0 GC showed a 5-year DSS of 98.9%, while a 5-year DSS of 95.1% and 90.0% were found for patients with pT1N1 GC and pT1N2 GC, respectively. Patients with pT1N3 GC showed a relatively poor prognosis with a 5-year DSS of 62.5% (**Figure 2A**).

**Table 3:** Univariate and multivariate analyses for the prognoses of early gastric cancer patients

Variables	5-year OS rate	Univariate	Multivariate		
		*P*	HR	95% CI	*P*
Age (years)		0.067			
< 60	97.50%				
≥ 60	95.40%				
Gender		0.678			
Male	96.60%				
Female	96.00%				
Tumor size (cm)		0.251			
≤ 2	96.80%				
> 2	95.50%				
Tumor location		0.68			
Upper	97.10%				
Middle	95.70%				
Lower	96.70%				
Gross type		0.329			
Ulcerated	95.60%				
Non-ulcerated	96.80%				
Degree of differentiation		0.098			
Differentiated	97.60%				
Undifferentiated	95.70%				
Histopathology		0.198			
Tub	97.60%				
Por	94.90%				
Sig	97.50%				
Muc	100%				
Pap	100%				
Examined lymph node		0.967			
Her-2 status		< 0.001*			
Negative	97.20%				
Positive	90.80%		3.082	1.512–6.281	0.002*
Depth of tumor invasion		< 0.001*			
T1a	98.60%				
T1b	94.20%		2.026	0.832–4.935	0.12
LNM status		< 0.001*			
Negative	98.30%				
Positive	84.80%		6.781	3.347–13.742	< 0.001*
Lymphovascular invasion		< 0.001*			
Negative	97.10%				
Positive	88.10%		1.359	0.605–3.054	0.458
Perineural invasion		< 0.001*			
Negative	96.60%				
Positive	75.00%		4.035	1.122–14.506	0.033*

*P < 0.05 was considered statistically significant. Tub, tubular adenocarcinoma; Por, poorly differentiated adenocarcinoma; Sig, signet-ring cell carcinoma; Muc, mucinous adenocarcinoma; Pap, papillary adenocarcinoma; T1a, mucosal invasion; T1b, submucosal invasion; OS, overall survival; HR, hazard ratio; CI, confidence interval.

#### Clinicopathological features and the effects of ACT on OS in pT1N1 GC patients

Among the 109 patients with pT1N1 GC, 46 (42.2%) patients underwent curative gastrectomy only (OBS group), while 63 (57.8%) received postoperative ACT (ACT group). Clinicopathological features of patients in the 2 groups are shown in **Table 4**. There was no statistically significant difference for any characteristics between the 2 groups, suggesting that all clinicopathological characteristics were comparable between the OBS and ACT groups. Survival analyses showed that there was no significant difference for the 5-year OS of pT1N1 patients in the OBS group (89.1%) and in the ACT group (90.5%) (*P* = 0.782) (**Figure 1B**). Univariate analyses for the prognoses of pT1N1 patients were performed next, and age, gender, tumor location, and Her-2 status had a statistically significant effect on the prognosis. Further multivariate analyses found that HER2 positive (HR: 4.517; 95% CI: 1.303–15.657; *P* = 0.017) and tumor middle location (HR: 3.761; 95% CI: 1.086–13.033; *P* = 0.037) were significant independent factors for the prognoses of pT1N1 GC patients (**Table 5**). Overall, 11 patients (10.1%) died within the 5-year follow-up, of which 5 patients died of tumor recurrence. Among the 5 patients, 3 patients received postoperative ACT and 2 patients underwent surgery only.

**Table 4:** Clinicopathological characteristics of patients with different treatment regimens

Variables (*n*, %)	pT1N1 (*n* = 109)			pT1N2 (*n* = 23)			pT1N3 (*n* = 19)		
	OBS (*n* = 46)	ACT (*n* = 63)	*P*	S-1 (*n* = 11)	Xelox (*n* = 12)	*P*	S-1 (*n* = 7)	Xelox (*n* = 12)	*P*
Age (years)			0.639			0.51			0.044*
< 60	24 (52.2)	30 (47.6)		7 (63.6)	6 (50.0)		7 (100)	6 (50.0)	
≥ 60	22 (47.8)	33 (52.4)		4 (36.4)	6 (50.0)		0	6 (50.0)	
Gender			0.735			0.214			0.656
Male	27 (58.7)	39 (61.9)		5 (45.5)	9 (75.0)		2 (28.6)	5 (41.7)	
Female	19 (41.3)	24 (38.1)		6 (54.5)	3 (25.0)		5 (71.4)	7 (58.3)	
Tumor size (cm)			0.92			0.059			0.96
≤ 2	26 (56.5)	35 (55.6)		8 (72.7)	4 (33.3)		4 (57.1)	7 (58.3)	
> 2	20 (43.5)	28 (44.4)		3 (27.3)	8 (66.7)		3 (42.9)	5 (41.7)	
Tumor location			0.334			0.775			0.731
Upper	2 (4.3)	4 (6.3)		2 (18.2)	1 (8.3)		0	1 (8.3)	
Middle	10 (21.7)	21 (33.3)		3 (27.3)	4 (33.3)		3 (42.9)	5 (41.7)	
Lower	34 (73.9)	38 (60.3)		6 (54.5)	7 (58.3)		4 (57.1)	6 (50.0)	
Gross type			0.177			0.4			0.35
Ulcerated	21 (45.7)	37 (58.7)		3 (27.3)	6 (50.0)		2 (28.6)	7 (58.3)	
Non-ulcerated	25 (54.3)	26 (41.3)		8 (72.7)	6 (50.0)		5 (71.4)	5 (41.7)	
Differentiation			0.302			0.155			0.433
Differentiated	6 (13.0)	13 (20.6)		1 (9.1)	5 (41.7)		0	1 (8.3)	
Undifferentiated	40 (87.0)	50 (79.4)		10 (90.9)	7 (58.3)		7 (100)	11 (91.7)	
Histopathology			0.63			0.116			0.432
Tub	6 (13.0)	13 (20.6)		1 (9.1)	5 (41.7)		0	1 (8.3)	
Por	32 (69.6)	42 (66.7)		8 (72.7)	6 (50.0)		6 (85.7)	7 (58.3)	
Sig	6 (13.0)	7 (11.1)		2 (18.2)	0		1 (14.3)	4 (33.3)	
Muc	2 (4.3)	1 (1.6)		0	1 (8.3)		0	0	
Examined lymph node	21 (17,29)	22 (18,34)	0.232	27 (19,31)	28 (21,35)	0.487	30 (19,34)	30 (17,43)	0.902
Her-2 status			0.061			0.901			0.683
Negative	43 (93.5)	51 (81.0)		8 (72.7)	9 (75.0)		6 (85.7)	11 (91.7)	
Positive	3 (6.5)	12 (19.0)		3 (27.3)	3 (25.0)		1 (14.3)	1 (8.3)	
Depth of invasion			0.889			0.052			0.433
T1a	10 (21.7)	13 (20.6)		3 (27.3)	0		0	1 (8.3)	
T1b	36 (78.3)	50 (79.4)		8 (72.7)	12 (100)		7 (100)	11 (91.7)	
Lymphovascular invasion	3 (6.5)	11 (17.5)	0.092	2 (18.2)	5 (41.7)	0.371	5 (71.4)	5 (41.7)	0.35
Perineural invasion	1 (2.2)	1 (1.6)	0.822	0	0	1	2 (28.6)	2 (16.7)	0.603

*P < 0.05 was considered statistically significant. Tub, tubular adenocarcinoma; Por, poorly differentiated adenocarcinoma; Sig, signet-ring cell carcinoma; Muc, mucinous adenocarcinoma; Pap, papillary adenocarcinoma; T1a, mucosal invasion; T1b, submucosal invasion; OBS, observation; ACT, adjuvant chemotherapy.

**Table 5:** Univariate and multivariate analyses for the prognoses of pT1N1 gastric cancer patients

Variables	5-year OS rate	Univariate	Multivariate		
		*P*	HR	95% CI	*P*
Age (years)		0.005*			
< 60	98.10%				
≥ 60	81.80%		6.491	0.805–52.325	0.079
Gender		0.031*			
Male	84.80%		5.103	0.648–40.176	0.122
Female	97.70%				
Tumor size (cm)		0.168			
≤ 2	93.40%				
> 2	85.40%				
Tumor location		0.027*			
Upper	100%				
Middle	77.40%		3.761	1.086–13.033	0.037*
Lower	94.40%				
Gross type		0.906			
Ulcerated	89.70%				
Non-ulcerated	90.20%				
Degree of differentiation		0.358			
Differentiated	84.20%				
Undifferentiated	91.10%				
Histopathology		0.486			
Tub	84.20%				
Por	89.20%				
Sig	100%				
Muc	100%				
Examined lymph node		0.644			
Her-2 status		0.015*			
Negative	92.60%				
Positive	73.30%		4.517	1.303–15.657	0.017*
Depth of tumor invasion		0.308			
T1a	95.70%				
T1b	88.40%				
Lymphovascular invasion		0.682			
Negative	89.50%				
Positive	92.90%				
Perineural invasion		0.642			
Negative	89.70%				
Positive	100%				
Treatment after surgery		0.782			
OBS	89.10%				
ACT	90.50%				

*P < 0.05 was considered statistically significant. Tub, tubular adenocarcinoma; Por, poorly differentiated adenocarcinoma; Sig, signet-ring cell carcinoma; Muc, mucinous adenocarcinoma; T1a, mucosal invasion; T1b, submucosal invasion; OS, overall survival; HR, hazard ratio; CI, confidence interval; OBS, observation; ACT, adjuvant chemotherapy.

In all, 103 patients were included in the DSS analysis after excluding 6 patients who died of other causes. Of these, 43 (41.7%) patients underwent curative gastrectomy only, while 60 (58.3%) patients received postoperative ACT. All clinicopathological characteristics were comparable between the OBS and ACT groups (all, *P* > 0.05) (**Supplementary Table S1**). The 5-year DSS of pT1N1 patients in the OBS group was 95.3% while in the ACT group it was 95.0%, with no significant difference found (*P* = 0.943) (**Figure 2B**). Univariate analyses showed that age and HER2 status had a statistically significant effect on the prognosis, and only HER2 positive (HR: 20.523; 95% CI: 2.281–184.687; *P* = 0.007) were significant independent factors of prognoses for pT1N1 GC patients using multivariate analyses (**Supplementary Table S2**).

#### Clinicopathological characteristics and the effects of ACT on OS in pT1N2 GC patients

All 23 pT1N2 GC patients received postoperative ACT, and 11 (47.8%) patients received S-1 monotherapy, while 12 (52.2%) received Xelox. Clinicopathological characteristics are shown in **Table 4**. There was no statistically significant difference for all characteristics between the S-1 and Xelox groups, indicating that all clinicopathological characteristics were comparable. Survival analyses indicated that the 5-year OS for patients in the S-1 group (81.8%) and in the Xelox group (91.7%) were not significantly different (**Figure 1C**) (*P* = 0.528). Using univariate analyses for clinicopathological characteristics with prognoses, no variables were found to have a significant effect on the prognosis (**Table 6**). Among the 23 pT1N2 patients, a total of 3 patients died during the 5-year follow-up. Two (1 patient received S-1 and 1 received Xelox) out of the 3 patients died of tumor recurrences.

**Table 6:** Univariate analyses for the prognoses of pT1N2 gastric cancer patients

Variables	5-year OS rate	*P*
Age (years)		0.427
< 60	92.30%	
≥ 60	80.00%	
Gender		0.304
Male	92.90%	
Female	77.80%	
Tumor size (cm)		0.459
≤ 2	91.70%	
> 2	81.80%	
Tumor location		0.555
Upper	66.70%	
Middle	85.70%	
Lower	92.30%	
Gross type		0.149
Ulcerated	100%	
Non-ulcerated	78.60%	
Degree of differentiation		0.815
Differentiated	83.30%	
Undifferentiated	98.20%	
Histopathology		0.923
Tub	83.30%	
Por	85.70%	
Sig	100%	
Muc	100%	
Examined lymph node		0.936
Her-2 status		0.815
Negative	88.20%	
Positive	83.30%	
Depth of tumor invasion		0.491
T1a	100%	
T1b	85.00%	
Lymphovascular invasion		0.147
Negative	93.80%	
Positive	71.40%	
Treatment after surgery		0.528
S-1	81.80%	
Xelox	91.70%	

Tub, tubular adenocarcinoma; Por, poorly differentiated adenocarcinoma; Sig, signet-ring cell carcinoma; Muc, mucinous adenocarcinoma; T1a, mucosal invasion; T1b, submucosal invasion; OS, overall survival.

In all, 22 patients were included in DSS analysis after excluding 1 patient who died of other causes. Of these, 10 (45.5%) patients received S-1 monotherapy while 12 (54.5%) received Xelox after curative resection. Clinicopathological characteristics were comparable between the S-1 and Xelox groups, except for tumor size. Patients with tumor size > 2 cm were more likely to receive Xelox (**Supplementary Table S1**). In addition, the 5-year DSS was 90.0% for pT1N2 patients in the S-1 group and 91.7% in the Xelox group (*P* = 0.922) (**Figure 2C**). Lymphovascular invasion was significantly associated with the prognosis using univariate analyses (*P* = 0.031) (**Supplementary Table S3**).

#### Clinicopathological characteristics and the effects of ACT on OS in pT1N3 GC patients

There were 19 patients with pT1N3 GC, and all of them received postoperative ACT. Patients who received S-1 monotherapy accounted for 47.8% (*n* = 7), while patients who received Xelox accounted for 52.2% (*n* = 12). Clinicopathological characteristics are shown in **Table 4**. Except for age (*P* = 0.044), other clinicopathological characteristics were comparable between the S-1 and Xelox groups (all, *P* > 0.05). Patients ≥ 60 years of age all received Xelox. Survival analyses showed no significant difference for the 5-year OS between the S-1 (14.3%) and Xelox groups (75.0%) (*P* = 0.009) (**Figure 1D**). The 5-year OS of pT1N3a patients and pT1N3b patients were 53.3% and 50.0%, respectively, with no significant difference found (*P* = 0.837). Only the postoperative therapy regimen was found to have a significant effect on prognosis using univariate analyses (**Table 7**). During the 5-year follow-up of the 19 pT1N3 GC patients, 9 patients died, of which 6 patients (4 patients received S-1 and 2 received Xelox) died of tumor recurrences.

**Table 7:** Univariate analyses for the prognoses of pT1N3 gastric cancer patients

Variables	5-year OS rate	*P*
Age (years)		0.418
< 60	46.20%	
≥ 60	66.70%	
Gender		0.603
Male	57.10%	
Female	50.00%	
Tumor size (cm)		0.514
≤ 2	45.50%	
> 2	62.50%	
Tumor location		0.404
Upper	100%	
Middle	37.50%	
Lower	60.00%	
Gross type		0.578
Ulcerated	44.40%	
Non-ulcerated	60.00%	
Degree of differentiation		0.413
Differentiated	100%	
Undifferentiated	50.00%	
Histopathology		0.412
Tub	100%	
Por	53.80%	
Sig	40.00%	
Examined lymph node		0.604
Her-2 status		0.669
Negative	52.90%	
Positive	50.00%	
Depth of tumor invasion		0.413
T1a	100%	
T1b	50.00%	
N stage		0.837
N3a	53.30%	
N3b	50.00%	
Lymphovascular invasion		0.287
Negative	66.70%	
Positive	40.00%	
Perineural invasion		0.114
Negative	60.00%	
Positive	25.00%	
Treatment after surgery		0.009*
S-1	14.30%	
Xelox	75.00%	

*P < 0.05 was considered statistically significant. Tub, tubular adenocarcinoma; Por, poorly differentiated adenocarcinoma; Sig, signet-ring cell carcinoma; T1a, mucosal invasion; T1b, submucosal invasion; OS, overall survival.

After excluding the 3 patients who died of other causes, 16 patients with pT1N3 GC were included in the DSS analysis. Among them, 5 (31.2%) patients received S-1 monotherapy, while 11 (68.8%) received Xelox after curative resections. There was no significant difference in clinicopathological characteristics between the S-1 and Xelox groups, except for lymphovascular invasion (**Supplementary Table S1**). All patients in the S-1 group had lymphovascular invasion, while 4 patients (36.4%) had lymphovascular invasion in the Xelox group (*P* = 0.034). The 5-year DSS was 20.0% for pT1N3 patients in the S-1 group and 81.8% in the Xelox group (*P* = 0.030) (**Figure 2D**). In addition, the 5-year DSS of pT1N3a and pT1N3b patients were 66.70% and 50.00%, respectively. Using univariate analyses, treatment regimens after surgery were found to have a statistically significant effect on the prognosis (*P* = 0.030) (**Supplementary Table S4**).

## Discussion

Perioperative complications and long-term gastrointestinal dysfunction may occur after radical gastrectomy with D2 lymphadenectomy. Endoscopic treatment has recently become an alternative therapy for EGC with a low risk of LNM due to its minimal invasiveness and preservation of function. The key to effective application of endoscopic techniques for radical gastrectomy of EGC is to accurately evaluate the risk of LNM^20^. In the present study, LNM occurred in 14.4% of EGC patients, and incidence of LNM was 5.3% and 23.0% for T1a tumors and T1b tumors, respectively. Similarly, Li et al.^21^ showed the incidence of LNM was 12.3% in EGC patients, and LNM in 13.3% of patients in the study by Pereira et al.^22^. One study reported the incidence of LNM as 2%–5% in EGC patients limited to the mucosa, while the incidence of LNM increased to 10%–25% in EGC patients with invasion of the submucosa^20^. We found that tumors greater than 2 cm in size, ulcerations, no differentiation, submucosa invasions, and lymphovascular invasions were more likely to occur with LNM, and tumor sizes > 2 cm, ulcerations, submucosa invasions, lymphovascular invasions, and no differentiation were independent risk factors for LNM. These results were confirmed in previous studies^21,23^. Endoscopic resection is therefore recommended for the treatment of differentiated and non-ulcerated tumors confined to the mucosa with sizes less than 2 cm. Thus, the accurate prediction of LNM could contribute to reducing the risk of overtreatment and unnecessary surgical complications.

Regarding long-term outcomes, EGC had a favorable prognosis with 5-year OS up to 96.4% in our study. In particular, the 5-year OS of pT1N0 patients was 98.3%, while it dramatically decreased in patients with LNM. Previous studies have found that EGC patients with LNM had a high recurrence compared to tumors without LNM, and LNM was an independent risk factor for recurrence^7,8^, which suggested that patients with LNM would benefit from ACT. Because there is no consensus between the Japanese and NCCN guidelines for the role of ACT in EGC, surgeons face a dilemma when deciding whether to administer ACT after curative surgery, especially in pT1N1 GC patients. We therefore evaluated the efficiency of different treatment regimens for pT1N1, pT1N2, and pT1N3 GC patients.

We found that the OBS and ACT groups showed similar 5-year OS and DSS for pT1N1 GC patients (*P* > 0.05), suggesting that ACT involved no survival benefit in pT1N1 GC patients. We further found that HER2 positive and middle tumor location were significant independent factors for the prognoses of pT1N1 GC patients. Consistently, based on the investigation of 510 pT1N1 GC patients in the Republic of Korea, Shin et al.^24^ found that patients in the surgery-only and ACT groups showed similar 5-year disease-free survival (DFS) (*P* > 0.05), and ACT had no benefit on tumor recurrence. Kim et al.^25^ evaluated 738 patients with pT1N1 GC in the Republic of Korea, reporting that the 5-year DFS of patients receiving surgery only (96.5%), ACT (96.0%), and adjuvant chemoradiotherapy (95.8%) showed no significant difference (*P* > 0.05), and both ACT and adjuvant chemoradiotherapy showed no benefits with respect to tumor recurrence. We therefore suggest that ACT might be unnecessary for pT1N1 GC after curative resection. However, in a retrospective analysis in a Western patient population (*n* = 696), Hester et al.^26^ reported that pT1N1 patients receiving adjuvant therapy had a median OS of 9.1 years, which was longer than patients receiving surgery only (4.6 years; *P* < 0.001). The effect of ACT in pT1N1 GC showed differences between Eastern and Western populations; thus, further studies are necessary to examine the pT1N1 GC subgroups that might benefit from adjuvant treatments.

Based on the 8th UICC/AJCC TNM staging system for GC patients, pT1N2 belongs to stage IIA, pT1N3a belongs to stage IIB, and pT1N3b belongs to IIIB. In the present study, the 5-year OS of pT1N2, pT1N3a, and pT1N3b patients were 87.0%, 53.3%, and 50.0%, respectively. In addition, the 5-year DSS of pT1N2, pT1N3a, and pT1N3b patients were 90.0%, 66.7%, and 50.0%, respectively. Thus, we combined pT1N3a and pT1N3b into the pT1N3 group.

According to the ACTS-GC trial^27^, a smaller number of patients (< 5%) in the S-1 group showed grade 3/4 adverse events, apart from anorexia, which occurred in 6% of the patients. However, the CLASSIC trial^28^ revealed more frequent grade 3/4 adverse events, of which 22% patients had neutropenia, and 8% had thrombocytopenia in the Xelox group. In our study, regarding pT1N2 GC, the prognosis in the S-1 group was poorer in comparison with the Xelox group, with a 5-year OS of 81.8% *vs.* 91.7%, but with no statistical significance (*P* = 0.528). In addition, patients in the S-1 group showed a 5-year DSS of 90.0%, which was 91.7% in the Xelox group, although there was no significant difference (*P* = 0.922). Considering adverse events, S-1 monotherapy is therefore considered a better choice for patients with pT1N2 GC.

Postoperative S-1 monotherapy significantly improved the survival of stage II/III GC patients who suffered curative surgery in the ACTS-GC trial^27^. Based on this result, adjuvant S-1 monotherapy for 1 year for stage II/III patients who had curative resection was recommended as a standard treatment. Nevertheless, stage IIIB patients showed a 5-year OS of 50.2% in the S-1 group, while the 5-year OS was 44.1% for patients who received surgery only in the ACTS-GC trial^14^, suggesting that S-1 exerted a weak effect in stage IIIB patients. Therefore, more intensive chemotherapy might be effective. In the CLASSIC trial^15^, stage IIIB patients had a 5-year OS of 66% in the Xelox group, while the 5-year OS was 45% for patients who received surgery only, indicating that adjuvant Xelox showed effects in patients in the Republic of Korea. In the present study, the Xelox group showed a significantly better prognosis when compared with the S-1 group (5-year OS: 75.0% *vs.* 14.3%; *P* = 0.009; 5-year DSS: 81.8% *vs.* 20.0%; *P* = 0.030) in patients with pT1N3 GC, showing that double chemotherapy exerted a stronger effect than monotherapy in adjuvant therapy. Thus, doublet chemotherapy is suggested for pT1N3 GC patients after curative resection.

In the present study, we systematically reviewed the role of ACT in EGC patients, and conducted subgroup analyses to identify the optimal postoperative adjuvant treatments for different N stages according to the UICC/AJCC TNM staging system for GC (8th edition). Based on our results, we conducted a trial to characterize the role of trastuzumab in HER2 positive EGC patients. Trastuzumab plus chemotherapy showed improved survival of HER2 positive advanced patients, according to the ToGA trial^29^.

Although our study comprehensively evaluated the effect of ACT in pT1 GC patients, there were some limitations. First, the study was retrospective, not prospective, and only patients who suffered surgical resection were enrolled. Patients who received endoscopic treatment should also be investigated. Second, the patients were from a single center, which might have led to selection bias. Third, we only analyzed OS and DSS, so the adjuvant treatment for recurrence of EGC also needs further evaluation. Fourth, adverse events of chemotherapy were not recorded. Finally, the number of patients with pT1N3 in this study was small, which may cause deviation of the results. Hence, prospective studies with large sample size are needed to validate the conclusions of this study.

## Conclusions

A positive LNM was correlated with worse prognoses in EGC patients. For patients with pT1N1 GC, observation after curative resection was adequate. S-1 monotherapy might be the optimal choice for pT1N2 GC patients, considering possible adverse events. Adjuvant doublet chemotherapy is therefore recommended for pT1N3 GC patients for better survival outcomes.
