# Supplementary material for: Is adjuvant chemotherapy necessary for early gastric cancer?
Source: Cancer Biol Med. 2021 Jun 23;19(4):518–32. doi: 10.20892/j.issn.2095-3941.2020.0636 (PMC9088187; doi:10.20892/j.issn.2095-3941.2020.0636)
Supplement: Supplementary file 1 [file cbm-19-518-s001.pdf]

Supplementary materials

Table S1 Clinicopathological characteristics of patients (except for patients who died from other factors) with different treatment regimens

Variables (*n*, %)	pT1N1 (*n* = 103)			pT1N2 (*n* = 22)			pT1N3 (*n* = 16)		
	OBS (*n* = 43)	ACT (*n* = 60)	*P*	S-1 (*n* = 10)	Xelox (*n* = 12)	*P*	S-1 (*n* = 5)	Xelox (*n* = 11)	*P*
Age (years)			0.550			0.415			0.119
< 60	24 (55.8)	29 (48.3)		7 (70.0)	6 (50.0)		5 (100)	6 (54.5)	
≥ 60	19 (44.2)	31 (51.7)		3 (30.0)	6 (50.0)		0	5 (45.5)	
Gender			0.690			0.192			0.513
Male	24 (55.8)	36 (60.0)		4 (40.0)	9 (75.0)		1 (20.0)	4 (36.4)	
Female	19 (44.2)	24 (40.0)		6 (60.0)	3 (25.0)		4 (80.0)	7 (63.6)	
Tumor size (cm)			0.882			0.043*			0.590
≤ 2	25 (58.1)	34 (56.7)		8 (80.0)	4 (33.3)		2 (40.0)	6 (54.5)	
> 2	18 (41.9)	26 (43.3)		2 (20.0)	8 (66.7)		3 (60.0)	5 (45.5)	
Tumor location			0.267			0.729			0.732
Upper	2 (4.7)	4 (6.7)		2 (20.0)	1 (8.3)		0	1 (9.1)	
Middle	8 (18.6)	19 (31.7)		3 (30.0)	4 (33.3)		2 (40.0)	5 (45.5)	
Lower	33 (76.7)	37 (61.7)		5 (50.0)	7 (58.3)		3 (60.0)	5 (45.5)	
Gross type			0.229			0.415			0.590
Ulcerated	20 (46.5)	36 (60.0)		3 (30.0)	6 (50.0)		2 (40.0)	6 (54.5)	
Non-ulcerated	23 (53.5)	24 (40.0)		7 (70.0)	6 (50.0)		3 (60.0)	5 (45.5)	
Differentiation			0.293			0.162			0.486
Differentiated	5 (11.6)	13 (21.7)		1 (10.0)	5 (41.7)		0	1 (9.1)	
Undifferentiated	38 (88.4)	47 (78.3)		10 (90.0)	7 (58.3)		5 (100)	10 (90.9)	
Histopathology			0.494			0.132			0.575
Tub	5 (11.6)	13 (21.7)		1 (10.0)	5 (41.7)		0	1 (9.1)	
Por	30 (69.8)	39 (65.0)		8 (70.0)	6 (50.0)		4 (80.0)	6 (54.5)	
Sig	6 (14.0)	7 (11.7)		2 (20.0)	0		1 (20.0)	4 (36.4)	
Muc	2 (4.7)	1 (1.7)		0	1 (8.3)		0	0	
Her-2 status			0.065			0.793			0.541
Negative	40 (93.0)	48 (80.0)		7 (70.0)	9 (75.0)		4 (80.0)	10 (90.9)	
Positive	3 (7.0)	12 (20.0)		3 (30.0)	3 (25.0)		1 (20.0)	1 (9.1)	
Depth of invasion			0.928			0.078			0.486
T1a	9 (20.9)	13 (21.7)		3 (30.0)	0		0	1 (9.1)	
T1b	34 (79.1)	47 (78.3)		7 (70.0)	12 (100)		5 (100)	10 (90.9)	
Lymphovascular invasion	3 (7.0)	11 (18.3)	0.097	2 (20.0)	5 (41.7)	0.381	5 (100)	4 (36.4)	0.034*
Perineural invasion	1 (2.3)	1 (1.7)	0.811	0	0	1.000	2 (40.0)	2 (18.2)	0.547

*$P < 0.05$ was considered statistically significant. Tub, tubular adenocarcinoma; Por, poorly differentiated adenocarcinoma; Sig, signet-ring cell carcinoma; Muc, mucinous adenocarcinoma; Pap, papillary adenocarcinoma; T1a, mucosal invasion; T1b, submucosal invasion; DSS, disease-specific survival; OBS, observation; ACT, adjuvant chemotherapy.

Table S2 Univariate and multivariate analyses for the prognoses of pT1N1 gastric cancer patients

Variables	5-year DSS rate	Univariate	Multivariate		
		P	HR	95% CI	*P*
Age (years)		0.019*			
< 60	100%				
≥ 60	90.00%		193834.2	0.000–2.599E+198	0.957
Gender		0.311			
Male	93.30%				
Female	97.70%				
Tumor size (cm)		0.42			
≤ 2	96.60%				
> 2	93.20%				
Tumor location		0.206			
Upper	100%				
Middle	88.90%				
Lower	97.10%				
Gross type		0.241			
Ulcerated	92.90%				
Non-ulcerated	97.90%				
Degree of differentiation		0.172			
Differentiated	88.90%				
Undifferentiated	96.50%				
Histopathology		0.497			
Tub	88.90%				
Por	95.70%				
Sig	100%				
Muc	100%				
Her-2 status		< 0.001*			
Negative	98.90%				
Positive	73.30%		20.523	2.281–184.687	0.007*
Depth of tumor invasion		0.238			
T1a	100%				
T1b	93.80%				
Lymphovascular invasion		0.691			
Negative	95.50%				
Positive	92.90%				

Table S2 Continued

Variables	5-year DSS rate	Univariate	Multivariate		
		P	HR	95% CI	*P*
Perineural invasion		0.751			
Negative	95%				
Positive	100%				
Treatment after surgery		0.943			
OBS	95.30%				
ACT	95.00%				

*$P < 0.05$ was considered statistically significant. Tub, tubular adenocarcinoma; Por, poorly differentiated adenocarcinoma; Sig, signet-ring cell carcinoma; Muc, mucinous adenocarcinoma; Pap, papillary adenocarcinoma; T1a, mucosal invasion; T1b, submucosal invasion; DSS, disease-specific survival; OBS, observation; ACT, adjuvant chemotherapy.

Table S3 Univariate analyses for the prognoses of pT1N2 gastric cancer patients

Variables	5-year DSS rate	*P*
Age (years)		0.816
< 60	92.30%	
≥ 60	88.90%	
Gender		0.08
Male	100%	
Female	77.80%	
Tumor size (cm)		0.868
≤ 2	91.70%	
> 2	90.00%	
Tumor location		0.198
Upper	66.70%	
Middle	85.70%	
Lower	100%	
Gross type		0.23
Ulcerated	100%	
Non-ulcerated	84.60%	
Degree of differentiation		0.486
Differentiated	83.30%	
Undifferentiated	93.80%	
Histopathology		0.885
Tub	83.30%	
Por	92.30%	
Sig	100%	
Muc	100%	
Her-2 status		0.486
Negative	93.80%	
Positive	83.30%	
Depth of tumor invasion		0.569
T1a	100%	
T1b	89.50%	
Lymphovascular invasion		0.031*
Negative	100%	
Positive	71.40%	
Treatment after surgery		0.922
S-1	90.00%	
Xelox	91.70%	

*$P < 0.05$ was considered statistically significant. Tub, tubular adenocarcinoma; Por, poorly differentiated adenocarcinoma; Sig, signet-ring cell carcinoma; Muc, mucinous adenocarcinoma; Pap, papillary adenocarcinoma; T1a, mucosal invasion; T1b, submucosal invasion; DSS, disease-specific survival; OBS, observation; ACT, adjuvant chemotherapy.

Table S4 Univariate analyses for the prognoses of pT1N3 gastric cancer patients

Variables	5-year DSS rate	*P*
Age (years)		0.418
< 60	54.50%	
≥ 60	80.00%	
Gender		0.418
Male	80.00%	
Female	54.50%	
Tumor size (cm)		0.957
≤ 2	62.50%	
> 2	62.50%	
Tumor location		0.228
Upper	100%	
Middle	42.90%	
Lower	75.00%	
Gross type		0.366
Ulcerated	50.00%	
Non-ulcerated	75.00%	
Degree of differentiation		0.481
Differentiated	100%	
Undifferentiated	60.00%	
Histopathology		0.196
Tub	100%	
Por	70.00%	
Sig	40.00%	
Her-2 status		0.464
Negative	64.30%	
Positive	50.00%	
Depth of tumor invasion		0.481
T1a	100%	
T1b	60.00%	
N stage		0.537
N3a	66.70%	
N3b	50.00%	
Lymphovascular invasion		0.134
Negative	85.70%	
Positive	44.40%	
Perineural invasion		0.052
Negative	75.00%	
Positive	25.00%	
Treatment after surgery		0.030*
S-1	20.00%	
Xelox	81.80%	

*$P < 0.05$ was considered statistically significant. Tub, tubular adenocarcinoma; Por, poorly differentiated adenocarcinoma; Sig, signet-ring cell carcinoma; Muc, mucinous adenocarcinoma; Pap, papillary adenocarcinoma; T1a, mucosal invasion; T1b, submucosal invasion; DSS, disease-specific survival; OBS, observation; ACT, adjuvant chemotherapy.
